# Decentralization of Care for Adults with Congenital Heart Disease in the United States: A Geographic Analysis of Outpatient Surgery

**DOI:** 10.1371/journal.pone.0106730

**Published:** 2014-09-23

**Authors:** Bryan G. Maxwell, Thane G. Maxwell, Jim K. Wong

**Affiliations:** 1 Department of Anesthesiology and Critical Care Medicine, Johns Hopkins University School of Medicine, Baltimore, MD, United States of America; 2 Department of Social Science, Metropolitan State University, Saint Paul, MN, United States of America; 3 Department of Anesthesiology, Perioperative, and Pain Medicine, Stanford University, Stanford, CA, United States of America; CUNY, United States of America

## Abstract

**Background:**

Guidelines recommend that adults with congenital heart disease (CHD) undergo noncardiac surgery in regionalized centers of expertise, but no studies have assessed whether this occurs in the United States. We hypothesized that adults with CHD are less likely than children to receive care at specialized CHD centers.

**Methods:**

Using a comprehensive state ambulatory surgical registry (California Ambulatory Surgery Database, 2005–2011), we calculated the proportion of adult and pediatric patients with CHD who had surgery at a CHD center, distance to the nearest CHD center, and distance to the facility where surgery was performed.

**Results:**

Patients with CHD accounted for a larger proportion of the pediatric population (n = 11,254, 1.0%) than the adult population (n = 10,547, 0.07%). Only 2,741 (26.0%) adults with CHD had surgery in a CHD center compared to 6,403 (56.9%) children (p<0.0001). Adult CHD patients who had surgery at a non-specialty center (11.9±15.4 miles away) lived farther from the nearest CHD center (37.9±43.0 miles) than adult CHD patients who had surgery at a CHD center (23.2±28.4 miles; p<0.0001). Pediatric CHD patients who had surgery at a non-specialty center (18.0±20.7 miles away) lived farther from the nearest CHD center (35.7±45.2 miles) than pediatric CHD patients who had surgery at a CHD center (22.4±26.0 miles; p<0.0001).

**Conclusions:**

Unlike children with CHD, most adults with CHD (74%) do not have outpatient surgery at a CHD center. For both adults and children with CHD, greater distance from a CHD center is associated with having surgery at a non-specialty center. These results have significant public health implications in that they suggest a failing to achieve adequate regional access to specialized ACHD care. Further studies will be required to evaluate potential strategies to more reliably direct this vulnerable population to centers of expertise.

## Introduction

Improved survival has resulted in dramatic growth in the prevalence of adult congenital heart disease (ACHD) [1], [2]. As survivors of congenital heart disease (CHD) spend proportionately more of their lives as adults, the relative importance of health events other than cardiac surgery will increase, and health systems must be prepared to care for them during a full range of medical interventions. Prior studies have demonstrated that ACHD is a risk factor for adverse outcomes at the time of noncardiac surgery [3], and a large cross-sectional study of ACHD patients found that perioperative death is the third most common cause of mortality (after sudden cardiac death and progressive heart failure) [4].

American College of Cardiology/American Heart Association consensus guidelines recommend that ACHD patients should receive perioperative care in specialized ACHD centers. ACHD patients are addressed both as a special population at risk in the guidelines for evaluation of cardiovascular disease for all patients undergoing noncardiac surgery [5] and by a more focused set of guidelines for the care of ACHD patients (in which noncardiac surgery is discussed as a potentially high-risk event) [6]. These guidelines specifically establish the standard that preoperative evaluation, risk assessment, and surgery for ACHD patients should occur in regional centers because of access to congenital cardiology care, experienced surgeons and cardiac anesthesiologists. However, no studies have assessed the degree to which this actually occurs.

We used the largest statewide database of outpatient surgery in the United States to determine the proportion of adults and children with CHD who undergo surgery in CHD centers of expertise and test the hypothesis that adults are less likely than children to receive care in specialized centers. We also sought to quantify proximity to CHD centers and compare geographic patterns of care among those who receive care at specialized centers and those who do not.

## Materials and Methods

The Stanford University Institutional Review Board granted an exemption from review because this research uses publicly available, deidentified data. The California Office of Statewide Health Planning and Development Ambulatory Surgery Data (OSHPD-AS) file is a comprehensive, public dataset of outpatient surgery encounters consisting of one record for each time a patient is treated in a licensed ambulatory surgery center in California. Records are provided by OSHPD via the Medical Information Reporting for California System. We examined OSHPD-AS records from years 2005 through 2011.

### Cohort creation

We used International Classification of Diseases, Ninth Revision, Clinical Modification (ICD-9-CM) diagnosis codes to classify records based on presence or absence of CHD (745.x, 746.x, and 747.1-4), divided records into adult (≥18 years) or pediatric (<18 years) categories, and classified all patients as having surgery at a CHD center or non-specialty center. CHD centers (n = 14) were defined as institutions meeting criteria based on the availability of CHD experts (Table 1).

**Table 1:** Criteria for defining CHD centers of expertise (must have ≥1 criterion).

Institution	City	ACHD cardiology fellowship*	Pediatric cardiology fellowship†	ACHD cardiology program‡	Surgeon member of Congenital Heart Surgeons Society§
		(n = 1)	(n = 5)	(n = 12)	(n = 7)
University of California – San Francisco	San Francisco		Y	Y	Y
Stanford University	Palo Alto		Y	Y	Y
Kaiser Permanente Northern California	San Francisco			Y	
University of California – Davis	Sacramento			Y	
Children's Hospital of Los Angeles	Los Angeles		Y		Y
University of California – Los Angeles	Los Angeles	Y	Y	Y	
Scripps Clinic	La Jolla			Y	
Loma Linda University	Loma Linda			Y	Y
St. Joseph's Hospital	Orange			Y	Y
Keck Medical Center, University of Southern California	Los Angeles			Y	Y
Kaiser Permanente Southern California	Los Angeles			Y	
Cedars-Sinai Medical Center	Los Angeles			Y	
University of California – San Diego	San Diego		Y		
Rady Children's Hospital	San Diego			Y	Y

*International Society for Adult Congenital Heart Disease (ISACHD) fellowship directory. http://isachd.org/fellowship. Accessed December 16, 2013. ACHD  =  adult congenital heart disease.

† American Council of Graduate Medical Education program listing. https://www.acgme.org/ads/Public/Programs/Search. Accessed December 16, 2013.

‡ Practices registered as ACHD practices in the Adult Congenital Heart Association (ACHA)/ISACHD database. http://www.achaheart.org/home/clinic-directory.aspx. Accessed December 16, 2013.

§ Congenital Heart Surgeons Society member directory. http://chss.org/multimedia/files/Member-Directory.pdf. Accessed December 16, 2013.

Patient home location was identified by home zip code. Records without a complete 5-digit California zip code or sufficient age information were excluded from analysis. Facility location was determined from the OSPHD facility identification number and linked to a publicly available master database with facility addresses [7]. Each facility (n = 907) was mapped by street address, and the distances from the geographic midpoint of the patient's home zip code to the surgical facility and to the nearest CHD center were calculated.

### GIS analysis

All GIS analyses used the “D_North_American_1983” datum and the “GCS_North_American_1983” geographical coordinate system. We used 2010 Tiger Line Shapefiles for Zip Code Tabulation Areas (the most accurate geospatial representation of zip codes) from the US Census Bureau [8] to define the geographic polygon of each zip code. We used the ArcGIS “Feature to Point” tool (Arc Toolbox > Data Management > Features > Feature to Point) to calculate the centroids of each zip code and identify the latitude and longitude coordinates of each centroid. Latitude and longitude coordinates from the previously geocoded OSHPD facility street addresses were also converted to a shapefile using the same tool.

Distance from the patient's home zip code to the surgical facility was calculated by using the “XY to Line” tool (Arc Toolbox > Data Management > Features > XY to Line; line type  =  geodesic) to create a line between each patient's home zip code and the surgical facility where they received care, then calculate the distance between each pair of points by calculating the length of each line in miles (using Field Calculator and python script “!shape.length@miles!”).

Distance from patient's home zip code to nearest ACHD center of expertise was calculated in an identical fashion, with the addition of a step in which the ArcGIS “Generate Near Table” tool (Arc Toolbox > Analysis > Proximity > Generate Near Table), was used to identify the nearest ACHD center, with “near features” defined as our list of ACHD centers and the “find closest near feature” setting selected.

These distances were exported as a delimited text file and imported into SAS, where they were merged (1∶1 inner join on unique record ID number) with the original OSHPD-AS file for further analysis.

### Statistical analysis

Descriptive results are presented as number (percentage) or mean ± standard deviation, for categorical or continuous variables, respectively. Cohorts were compared using Fisher's exact test for categorical variables and the Mann-Whitney-Wilcoxon test for continuous variables. A predetermined alpha of 0.05 was used as the threshold of statistical significance. Geocoding (conversion of street addresses to precise latitude and longitude coordinates) was performed with the Google Maps Geocoding API (version 3, Google, Inc, Mountain View, CA). GIS analyses were performed using ArcGIS (version 10.2.1, Esri, Redlands, CA). Statistical analyses were performed using SAS (SAS 9.3, SAS Institute, Cary, NC).

## Results

Of a total of 18.1 million outpatient surgical encounters, 16.3 million (90.1%) had adequate patient age and home zip code information for analysis. The 21,801 CHD patients represented 0.13% of the overall study population. CHD was present in 1.0% (n = 11,254) of pediatric records and 0.07% (n = 10,547) of adult records.

Within the CHD population, adults were significantly less likely to receive care in a CHD center: 2,741 (26.0%) adults with CHD had surgery in a CHD center compared to 6,403 (56.9%) children with CHD (p<0.0001). For reference, CHD centers accounted for only 10.7% (n = 1.7 million) of encounters in the non-CHD population.

Adult CHD patients who had surgery at a non-specialty center lived farther from the nearest CHD center (37.9±43.0 miles) than adult CHD patients who had surgery at a CHD center (23.2±28.4 miles; p<0.0001). Adult CHD patients who had surgery at a non-specialty center traveled an average of 11.9±15.4 miles to their surgical facility (26.0±41.4 miles closer than the nearest CHD center). Among adult CHD patients who had surgery at a CHD center, 51.1% had surgery at a CHD center other than the nearest one, and traveled an excess of 11.2±20.2 miles to do so.

Pediatric CHD patients who had surgery at a non-specialty center lived farther from the nearest CHD center (35.7±45.2 miles) than pediatric CHD patients who had surgery at a CHD center (22.4±26.0 miles; p<0.0001). Pediatric CHD patients who had surgery at a non-specialty center traveled an average of 18.0±20.7 miles to their surgical facility (17.7±40.3 miles closer than the nearest CHD center). This was farther than the distance traveled by the equivalent adult subgroup to a non-specialty center (11.9±15.4 miles; p<0.0001).

**Figure 1:**
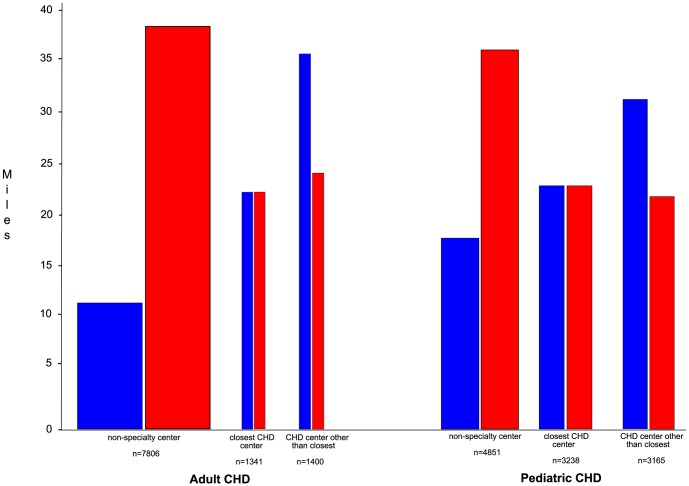
Distance from care. Values are the mean distance to the facility where surgery occurred (blue bars) and to the nearest CHD center of expertise (red bars), by patient subgroup. Width of each pair of columns is proportional to the relative size of the corresponding subgroup.

Among pediatric CHD patients who had surgery at a CHD center, 49.4% had surgery at a CHD center other than the one closest to them, and traveled an excess of 9.1±16.0 miles to do so. Figure 1 depicts the distances traveled by each of these patient subgroups.

## Discussion

The principal finding of this study is that adults with CHD are less likely than children with CHD to travel to a specialized center for outpatient noncardiac surgery. The majority of adults with CHD (74%) had surgery in a non-specialty center, whereas the majority of children with CHD (57%) received care at CHD centers of expertise.

These results suggest that in both the adult and pediatric CHD populations, distance is a barrier to accessing centers of expertise. This is consistent with prior data that distance is a risk factor for not receiving appropriate outpatient general pediatric care [9] or and that receiving care at a community versus referral hospital center predicts poorer access to specialist cardiology care [10]. Prior surveys of parental preferences have demonstrated that in hypothetical scenarios involving the choice between surgery at a local center and a referral hospital, greater proportions of families will choose the local center as the distance to the referral hospital increases, even if presented with the tradeoff that there is a higher mortality rate for surgery at the local center [11].

However, distance alone does not account for the large discrepancy in the proportion of adults compared to children who travel to a CHD center for care or the smaller subgroup of adults (13.3% compared to 28.1% of children) who traveled excess distance to a CHD center other than the one closest to them. This finding that surgical care for adults appears to be more decentralized than that for children is consistent with prior analysis demonstrating a more decentralized pattern of hospitalizations for adults versus children with CHD [12].

These results have significant public health implications for the adult CHD community, as they demonstrate that the majority of adult CHD patients do not receive what has been recommended as a standard of care by AHA/ACC guidelines: access to CHD centers of expertise for noncardiac surgery. The finding that adults are less likely to receive care in regional centers of expertise is even more striking when considering that the complexity of CHD is higher in the adult compared to the pediatric population.

Further studies should investigate barriers to meeting this standard. In addition to physical distance, we can speculate based on prior examinations of the place of ACHD care within the U.S. health care system that other factors may include a dearth of ACHD-specific cardiology providers, loss to follow-up at the time of graduation from pediatric care networks [13], inadequate insurance coverage, and suboptimal self-awareness of diminished physiologic reserve [14] and lifelong risk for patients who may regard themselves as healthy, normal adults who “used to have” a heart problem [15]. While challenges to achieving adequate access to care for ACHD patients have been encountered in many developed nations, even those with integrated health systems and universal health insurance (e.g. Germany [15], the Netherlands [16], and Canada [17]), many of these potential factors may be uniquely exacerbated in the American setting, where a large proportion of the population has no or inadequate health insurance, and even those with insurance face a fragmented system fraught with financial (e.g. high deductibles and co-payments) and administrative barriers (e.g. provider network restrictions, gatekeepers, prior authorization requirements) to receiving care.

Our study has several important limitations. Patient privacy concerns limit the granularity of geographical information to zip code [18]. The unquantifiable – but likely small – error introduced by the use of zip codes in lieu of precise street addresses is unlikely to systematically affect any subgroup disproportionately, and our study nonetheless was large enough to detect highly significant differences.

Our data source did not allow us to calculate one distance parameter that would have been ideal: the distance to the nearest surgical facility for patients who did have surgery at a CHD center (i.e. how much farther than the minimum distance did they travel for care?). Since many facilities in the OSHPD database are small office-based surgical centers, we could not use the closest facility of any type, as it would not be valid to assume that any procedure could be provided in any setting. We believe the converse assumption (that a CHD center is capable of providing the full range of surgical services available at smaller centers) is valid.

No consensus definition exists for what constitutes a CHD center; our criteria admittedly are subjective, but we consider them justified and non-arbitrary. We did not separately define adult and pediatric CHD centers, as some adults continue to receive care at pediatric facilities.

We selected California because it provides the largest contained state dataset and is less subject than other states (e.g. the northeastern U.S.) to inter-state travel to receive care. These results may approximate patterns of care outside California and for other types of health care beyond outpatient surgery, but the external validity of these results to those contexts cannot be established by this analysis.

Despite these limitations, this is the first study to analyze geographic patterns of access to outpatient surgical care in adults and children with CHD. It demonstrates that existing systems have not achieved the ideal of regionalized ACHD expertise in noncardiac surgery, and it provides support for policy and patient advocacy efforts to improve access to high-quality, specialty care for ACHD.
